# Percutaneous Endovascular Reconstruction of the Common Femoral Artery and Its Bifurcation

**DOI:** 10.3390/jcm13113169

**Published:** 2024-05-28

**Authors:** Stephanie Rassam, Raphaël Coscas

**Affiliations:** 1 Division of Vascular and Endovascular Surgery, Department of Heart, Vascular and Endovascular Surgery, Paracelsus Medical University, 5020 Salzburg, Austria; dr.stephanierassam@gmail.com; 2 Department of Vascular Surgery, Centre Hospitalier Universitaire Ambroise Paré, Assistance Publique Hôpitaux de Paris (AP-HP), 92104 Boulogne-Billancourt cedex, France; 3 UMR 1018, Inserm-Paris11—CESP, Versailles Saint-Quentin-en-Yvelines University, Paris-Saclay University, Paul Brousse Hospital, 94807 Villejuif, France

**Keywords:** peripheral arterial disease, endovascular procedures, femoral artery, vascular calcification

## Abstract

Occlusive lesions of the common femoral artery (CFA) and its bifurcation have traditionally been treated with open surgery. Although long-term patency rates after open surgery are excellent, such repairs are associated with substantial local and general morbidity. In recent years, different treatment options have emerged within percutaneous endovascular repair. We hereby present a narrative review on endovascular treatment modalities and a treatment algorithm for endovascular revascularisation of the CFA and its bifurcation. Lesion analysis, access issues, vessel preparation tools, and types of repairs with or without the involvement of the bifurcation are described. Based on current data, an interventional approach can result in high technical success and acceptable mid-term patency rates. Further comparative evidence with open surgery and/or between the different types of endovascular repairs is required to improve the current treatment algorithm.

## 1. Introduction

The common femoral artery (CFA) is the gateway for inflow and outflow in peripheral artery disease (PAD). However, occlusive lesions of the CFA and its bifurcation are frequent in daily practice. For decades, open surgical revascularisation (OSR) of the CFA, generally through an endarterectomy, has been viewed as the gold standard. Although long-term patency rates after OSR are excellent, such repairs are associated with substantial local and general morbidity. Over the past years, different percutaneous endovascular treatment (EVT) options have emerged as alternatives. The objective of this narrative review is to provide the reader with a better understanding of the EVT strategies used in CFA repair, specifically focusing on technical aspects.

## 2. Methods

A literature search was performed using PubMed, the Cochrane Library, and clinicaltrials.gov. The search terms included “common femoral artery” in combination with the Boolean operators AND or OR “endarterectomy”, “endovascular”, “angioplasty”, “stenosis”, “occlusion”, “stent”, “balloon”, “peripheral artery disease”. This was extended to related articles. Studies which analysed OSR or EVT of the CFA with or without its bifurcation were included, supplemented by comparative studies of these treatment options, periprocedural management, and studies on femoropopliteal repair. No studies were excluded based on their year of publication. No limits were set regarding study design. Studies written in English, German, or French were searched for inclusion.

## 3. Narrative Review

### 3.1. The Drawbacks of CFA Open Repair

Endarterectomy has historically been the treatment modality of choice for atherosclerotic lesions of the CFA. This has been justified by a perceived low risk of morbidity combined with high patency rates [1,2,3]. Upon closer inspection, OSR may not be as innocuous as originally believed, with postoperative complications delineated in up to one-quarter of treated patients. Research has been limited to registries and a small number of trials, which have not indicated a decrease in periprocedural complication rates over the past decades [3,4,5,6,7,8,9,10,11]. The most frequent complications are still related to the incision site and include wound complications owing to infection, dehiscence, lymphatic leakages, haematoma, and neural dysaesthesia [9,12,13]. Different factors associated with local postoperative complications have been depicted, comprising advanced age, diabetes mellitus, obesity, open surgical revision, and the use of drains [5,8,9]. Early mortality rates have been recurrently reported, with rates above 3% and with a perceived increase in emergency cases. They, too, have been linked to advanced age and to poor functional or physical status, end-stage renal disease requiring dialysis, congestive cardiomyopathy, and sepsis [4,6,12]. Since the PAD population presents at an advanced age and with various co-morbidities, a minimally invasive approach represents an attractive solution to overcome complications associated with OSR.

### 3.2. The Current Standpoint of Endovascular CFA Repair

EVT of the CFA has been considered controversial due to several challenges. One of the main issues is the high level of nodular calcification, which is often accompanied by osteoid metaplasia. This can limit lumen gain, which makes vessel preparation a necessity [14,15]. In advanced disease affecting the bifurcation, the challenge is reflected by the aim of preserving both the superficial (SFA) and the deep femoral arteries (DFA). Regarding stenting of the CFA, there is concern about durability as the zone to be treated is in proximity to the hip joint, with a subsequently believed risk of stent fracture. Data have not supported this hypothesis, with the zone in motion not primarily affecting the CFA [16,17]. Another concern is sacrificing a potential access site due to the stenting of the CFA. Treatment modalities and technical considerations have been explored to refute these aspects considering a needed minimally invasive approach in selected patients.

Until recently, guidelines of vascular surgical societies did not consider EVT of the CFA. In the European Society for Vascular Surgery (ESVS) guidelines published in 2017, EVT of the CFA was not an option [18]. According to the Global Vascular Guidelines (GVG) on the management of chronic limb-threatening ischaemia (CLTI), EVT can be considered in patients with a hostile groin or high-risk patients for OSR of the CFA (Class 2, Level C) [1]. As a good practice statement, stents are proposed to be avoided across the origin of a patent DFA. Due to similar mid-term patency, EVT may be considered alternatively to OSR in non-complex CFA lesions which do not extend down to the femoral bifurcation (Class IIb, Level B). No further details are delineated about the type of revascularisation strategy in this context. More recently, the 2024 ESVS guidelines on the management of intermittent claudication have supported EVT for extensive lesions affecting the CFA bifurcation in patients with a hostile groin or morbid obesity (Class IIb, Level C). For CFA stenosis or occlusion that does not affect the femoral bifurcation, EVT has been outlined as a viable alternative to OSR owing to similar mid-term patency rates in non-complex lesions (Class IIb, Level B) [2]. 

### 3.3. Preoperative Assessment

To thoroughly plan an EVT approach, pre-interventional imaging is a necessity. Imaging is initially performed via ultrasound, providing good information on vascular anatomical and morphological characteristics. It is a widely available diagnostic tool which has its limitations, being time-consuming and investigator-dependent. The preferred imaging method before revascularisation is computed tomography angiography, which aids in planning the intervention. This way, the appropriate angulation for adequate visualisation of the PFA can be priorly known, reducing fluoroscopy time and with it contrast medium load. Visualisation via magnetic resonance angiography has the downside of creating artefacts in calcified lesions or priorly deployed stents, which may lead to a wrong interpretation [19,20]. Visualisation can be optimised intraprocedurally with intravascular ultrasound (IVUS) [21,22]. 

### 3.4. Anatomical Depiction of the Disease

Lesions of the CFA vary in morphology and extension of disease. A standardised classification system allows for a better indication of revascularisation strategies and reporting in studies. Different classifications have so far been delineated. In 2011, two classification approaches were presented in the literature. The Medina classification used for coronary artery disease was modified and presented by Bonvini et al. to describe lesion location through a three-point binary schematic [23]. Azéma et al. classified CFA lesions into four groups in which extension of disease (groups I–III) and proximal or distal stenoses of bypass anastomoses (group IV) are included [24]. Recently, Rabellino et al. revised the Azéma classification by excluding stenotic bypass lesions and supplementing the given classification with morphological changes and affected adjacent areas of the native artery [25]. We agree with the importance of the supplemented morphological descriptions. The anatomic grading system presented within the Society of Vascular Surgery (SVS) reporting guidelines for endovascular treatment can help with this, in addition to better depicting the morphology of the disease [26]. 

### 3.5. Access

#### 3.5.1. General Aspects

Safe access is a necessity for EVT and should thoroughly be planned before the intervention, taking the sheath size and possible vascular closure device (VCD) into consideration. This emphasises the importance of having high-quality imaging before revascularisation. It is important to consider a bigger sheath size in cases of bifurcated repairs that are planned via a single sheath. This may necessitate sheath sizes up to 10 F, requiring a preclosure device as the VCD, which would in turn need partial deployment at an earlier stage of the procedure, or an open cut-down. Preprocedural planning of the approach is also important in cases of an upper-limb approach, as no VCDs have been approved to date [27]. 

**Figure 1:**
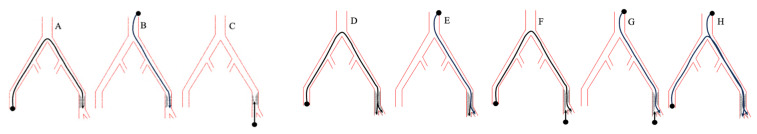
**Options for access to the common femoral artery**. Access for simple lesions can be obtained through a cross-over (**A**), upper-limb (**B**), or retrograde approach (**C**). Access for complex lesions is possible with one sheath through a cross-over (**D**) or an upper-limb approach (**E**); access with two sheaths is possible through a cross-over and retrograde (**F**), retrograde and upper-limb (**G**), and cross-over and an upper-limb approach (**H**).

For CFA repair, access can either be acquired through (1) the contralateral side with a cross-over, (2) the retrograde ipsilateral SFA, or (3) an upper-limb approach in various combinations (Figure 1). Via ultrasound, the optimal puncture site can easily be identified, enabling the visualisation of ventral wall disease in terms of atherosclerotic or thrombus burden. It may reduce the time and number of attempts to access in patients with a faint or absent pulse, overweight, or tissue alterations due to previous postprocedural complications. In addition, puncture of the femoral vein and other complications can be minimised [28,29]. Patient-related risk factors associated with local vascular complications are female sex, older age, overweight, underweight, uncontrolled hypertension, renal failure, and concurrent anticoagulation with increased international normalised ratio (INR). Procedure-related risk factors associated with local vascular complications are large sheath sizes, concomitant use of venous sheaths, location of the arteriotomy, previous entry at the same site, and prolonged sheath duration [30]. We prefer and recommend an ultrasound-guided approach.

#### 3.5.2. Contralateral Common Femoral Artery

For CFA access, the inguinal crease is commonly used as a reference area as it is believed to be related to the inguinal ligament. This can be true, but the distance can differ and be larger in female patients [16]. In cases of a patent contralateral CFA, a cross-over approach can be a feasible option for access. Data on rates of bilateral CFA lesions have not been delineated in the literature to date, but these may regularly appear, as atherosclerotic diseases are systemic conditions. The CFA lumen enables the usage of different sheath sizes, which may be helpful whenever a wide lumen is present in the contralateral CFA or in complex lesions. Another advantage is given by the ability to use short sheaths and shafts due to the limited route, which eases navigation. If access is warranted through the CFA, different VCDs can be utilised for closure. In malfunctioning cases or cases of complications, manual compression might be sufficient. If not, a cut-down can be performed to suture the access area on the CFA. If a high atherosclerotic burden is present, a thrombendarteriectomy with patch angioplasty will be needed and can be performed in this setting, or, and this is case-dependent, as a staged procedure.

#### 3.5.3. Ipsilateral Superficial Femoral Artery

In cases of contralateral CFA disease, a feasible alternative access point is presented by the ipsilateral SFA. This necessitates a stenotic-free and well-accessible area. Several studies have suggested that a retrograde approach through the ipsilateral SFA is a safe and reasonable option [31,32,33,34]. The SFA can be located via ultrasound. However, depending on the depth of its localisation, a longer puncture needle might be beneficial. The advantages of SFA access include the straight course of the SFA and its size, which is larger than a radial or brachial artery. Still, the size of the SFA needs to be priorly assessed to ensure that sheath insertion does not cause complications [32,33,34]. Complication rates are similar to those in CFA punctures. Haemostasis can be achieved either via manual compression or a vascular closure device (VCD), which is not inferior in its use in the SFA, though a diameter of above 5 mm will be necessary [34,35]. A disadvantage of this approach is the lack of a bony surface for effective compression in case of device failure. This aspect also impedes first-line manual compressions [33,34,35,36]. 

#### 3.5.4. Upper-Limb Approach

Approaches through the axillary, brachial, or radial artery represent additional access options in cases of bilateral CFA disease. They have shown to be a reliable and effective access approach in EVT and are regularly performed through the left side to avoid arch manipulation [37]. The brachial or axillary artery are preferred approaches in cases that require a large sheath insertion [24,38,39]. No VCDs have been accepted for use in the brachial artery. There have been trials reporting off-label use that suggest they may be safe. Further studies are necessary to evaluate the safety and efficacy of VCDs for the upper-limb approach.

#### 3.5.5. The Choice of Access

**Figure 2:**
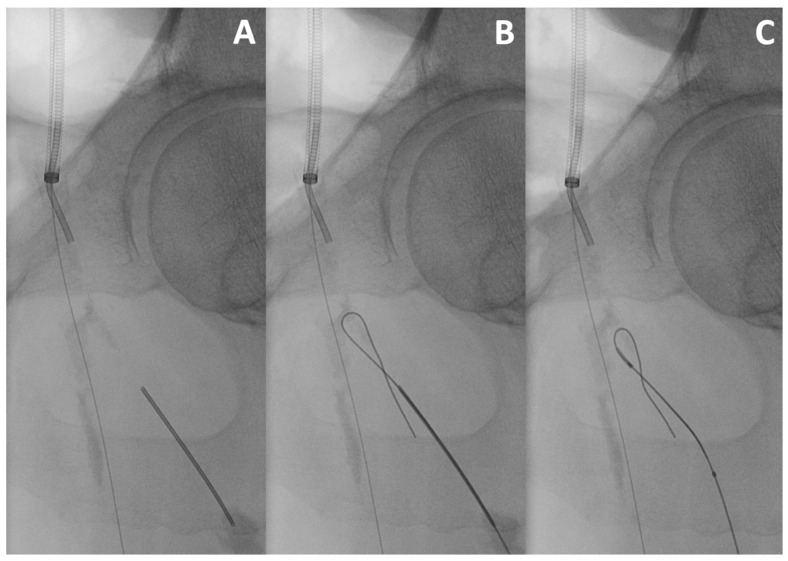
**Retrograde deep femoral artery access**. After sheath insertion into the common femoral artery via a cross-over approach, retrograde access via puncture of the deep femoral artery with a 21-Gauge micro-puncture needle is performed (**A**). The 0.018″ guidewire is advanced into the femoral bifurcation (**B**). A 0.018″ support catheter is placed, and the guidewire is further advanced in a looped manner to cross the lesion without sheath insertion (**C**).

For simple lesions, a cross-over or upper-limb approach can be chosen, with the alternative of retrograde access, as visualised in Figure 2. For complex lesions, approach considerations depend on the introducer that will be used, which again depends on the vessels’ morphology and patient’s characteristics. If only one access site is preferred or possible, a sheath measuring 8 French (Fr) or above can be used through a cross-over or upper-limb approach. If two sheaths are preferred, a 6 to 7 Fr sheath will be sufficient with access through either of the aforementioned sites.

### 3.6. Lesion Crossing

EVT is challenging due to the different characteristics of lesions with mixed plaque morphology, which may be accompanied by chronic total occlusions in either of the branches of the femoral artery. Different crossing techniques and devices may aid in penetrating and crossing lesions. In cases of a stenosis, an optimised perfusion can be easily achieved with simple techniques [40]. Appropriate guidewires for a lower limb revascularisation are the 0.035″ (primarily compatible with at least 6F sheath) and 0.018″ (commonly 4 or 5 Fr sheath) to 0.014″ (commonly 4 Fr sheath) guidewires. The larger the calibre of the guidewire is, the higher the supporting force, torque control, and pushability, while flexibility is reduced. Guidewires known for their crossing ability are characterised by a high penetration ability and adequate torque control of their tip [41]. 

#### 3.6.1. True Lumen Crossing Techniques

Due to the usually calcified nature of the lesion, endoluminal crossing can be more challenging than a sub-intimal approach in the CFA. The most used method is a catheter-assisted direct endoluminal technique. For this, a 0.018″ or 0.014″ hydrophilic guidewire with a flexible tip can be used to catheterise the residual true lumen, if present. Approximating the support catheter to the lesion can aid in supporting the wire to be able to intubate the lesion. As the CFA is prone to a higher calcification load, a perforating technique could be useful. For this, commonly non-hydrophilic guidewires with a high-load tapered tip are used, e.g., the Astato (ASAHI Intecc, Aichi, Japan) guidewire. If the technique results in a sub-intimal crossing without successful endoluminal re-entry, the used guidewire can be left in place. This way, the guidewire will not automatically enter the previously approached path. Instead, a new guidewire can be used, utilising the same technique. This can be performed with multiple wires. In femoral bifurcated stenosis, if the guidewire is repeatedly intubating the adjacent vessel due to a proximal occlusion of the vessel-to-treat, a balloon can be inflated at low pressure into the ostium of either the SFA or DFA. This will foreground concentrating on guidewire advancement into the affected vessel. Endoluminal entry can also be achieved with a different balloon-assisted technique. This technique requires the guidewire to be centrally arranged in a linear course of the vessel. This method should be initiated with a 0.014″ wire, which is light-tipped and can successively be intensified. Guidewire advancement can be facilitated by IVUS or two fluoroscopic projections. From experience, a different guidewire size has not proved to be safe for this crossing technique. If crossing cannot be achieved this way, this manoeuvre can be supplemented with an angled side catheter, to advance through another guidewire at a different angle. Other higher-risk techniques have been delineated but must be performed meticulously and with caution as they can result in perforation [40]. 

#### 3.6.2. Sub-Intimal Crossing Techniques

Sub-intimal crossing techniques may ease crossing through complex, long lesions and chronic total occlusion (CTO) of the CFA and its bifurcation. Generally, this technique might speedily enable crossing if adequate re-entry is possible. Re-entry catheters (e.g., BeBack, Bentley InnoMed GmbH, Hechingen, Germany) can help attain endoluminal passage but are not always required. The above-mentioned endoluminal techniques can be adapted to sub-intimal entries. Atherectomy should not be performed in such cases. The disadvantages of perforation and collateral branch occlusion should not be disregarded. This can result in injuries to the SFA or DFA. A dissection may further progress and ultimately result in occlusion of either or both vessels. Stenting will stabilise the intimal tear, which is why bail-out bifurcated stenting strategies must be known to be able to limit this risk [40]. 

#### 3.6.3. Retrograde Superficial or Deep Femoral Artery Puncture

This useful bail-out manoeuvre can be used when the SFA or the DFA cannot be catheterised through an antegrade approach. There is a paucity of data delineating retrograde deep femoral artery access, with first descriptions dating back to 1990 [25,42,43,44]. Retrograde SFA puncture can be easily performed under ultrasound-guidance and is as such not a challenge per se. DFA puncture is more difficult. It is generally performed under fluoroscopic guidance using a 21 or 22 Gauge micropuncture needle after positioning the C-arm in an ipsilateral oblique view. It can also be performed under ultrasound when its course is not too deep. A 0.018″ wire is then inserted over a support catheter to be captured and externalised in the antegrade access and allows for lesion crossing (Figure 2).

### 3.7. Vessel Preparation

The technical advances of the past decades have allowed us to add newer treatment modalities to our armamentarium. For preparation, different tools are available, varying in technical aspects and cost [26]. Speciality balloons may induce barotrauma to the vessel and dissection, necessitating the use of a stent thereafter. The choice of preparation tool and treatment will depend on the experience of the treating physician, the availability of vessel preparation tools, stenting devices, and reimbursement. While the cost of a simple balloon angioplasty will be above EUR 100 per balloon used, cutting balloons will cost octuple that. Regarding atherectomy or intravascular lithotripsy, prices go above EUR 2000- and cannot be guaranteed in every centre.

#### 3.7.1. Balloon Angioplasty

##### Plain Old Balloon Angioplasty (POBA)

The most common EVT used on limbs is plain old balloon angioplasty. With its characteristics, it is limited in cases of CFA vessel preparation owing to the known high calcification burden in the CFA. High-pressure and ultra-high-pressure balloons are more commonly used in arteriovenous-fistula stenosis but may represent an alternative treatment approach.

##### Peripheral Cutting Balloons (PCB)

The cutting balloon (Boston Scientific Corporation, Natick, MA, USA) is a peripheral cutting balloon, which has atherotomes attached. These microsurgical blades displace plaque upon inflation after mildly inflating the balloon to ensure plaque contact. PCBs with diameters ranging up to 8 mm can be used in the CFA.

##### Chocolate Percutaneous Angioplasty (PTA) Balloon

The Chocolate PTA Balloon (Medtronic, Minneapolis, MN, USA) represents a semi-compliant balloon that enables one-to-one vessel sizing. As the balloon is inflated, pillows will cause small dissections necessary for sufficient dilation, while grooves help prevent the dissections from propagating. The available diameter ranges up to 6 mm, making its usage limited to smaller vessels or for partial lumen gain prior to stenting.

##### Angiosculpt Balloon

The Angiosculpt Balloon (Philips Holding Inc., San Diego, CA, USA) is a semi-compliant scoring balloon, encircled by three to four helical nitinol struts. These struts create the luminal enlargement needed for stent implantation, which is commonly followed thereafter. With diameters ranging up to 8 mm, the Angiosculpt balloon can be utilised in the CFA.

#### 3.7.2. Atherectomy

##### General Concepts

There are different types of atherectomy devices used in endovascular repair that technically aim to mimic open surgery through plaque removal. Atherectomy can be a good option to debulk plaque while ensuring a “leave nothing behind” strategy. This is limited to the intima and can enable a smoother vascular bed with a reduction in stenosis grade. With atherectomy, soft tissue and calcification cannot be differentiated. Different atherectomy devices are currently on the market.

Directional atherectomy devices have a low-pressure threshold. This decreases the risk of dissection and postprocedural neo-intimal hyperplasia. They are preferred for eccentric plaque configurations, as regularly seen in the CFA. The SilverHawk^TM^ (Medtronic, Minneapolis, MN, USA) and the TurboHawk^TM^ Atherectomy Devices (Medtronic, Minneapolis, MN, USA) come in various sizes, enabling atherectomy in vessels up to 7 mm. TurboHawk^TM^ is preferred for highly calcified lesions owing to its four contoured blades, while SilverHawk^TM^ has one. With orbital atherectomy, larger vessels can be treated, using the provided high-speed feature without the need to upsize the catheter. In comparison to rotational atherectomy, it creates more calcium modification, produces noncalcified plaque modification, and does not need a filter for embolic protection. Full 360-degree contact with the vessel wall can be facilitated with the Diamondback 360^®^ (Abbott Vascular, Santa Clara, CA, USA) orbital atherectomy device. Rotational excisional atherectomy has a high-speed rotating device tip, e.g., the JetStream^TM^ (Boston Scientific Corporation, Natick, MA, USA), that circumferentially grinds plaque. Different benefits of existing atherectomy devices are combined in hybrid atherectomy devices, e.g., the Phoenix Atherectomy System^TM^ (Philips Holding Inc., San Diego, CA, USA), which enables cutting, capturing, and clearing soft plaque to calcification through one insertion. Ablative laser atherectomy reduces plaque load without removing it, e.g., the CVX-300 Excimer laser system (Philips, Holding Inc., San Diego, CA, USA). Despite the aforementioned advantages, atherectomy is viewed in an ambivalent way owing to the occurrence of periprocedural complications [44,45,46,47,48]. 

##### Technical Considerations

We decided not to elaborate on the technical considerations of each atherectomy device, as this would go beyond the scope of this review. An important consideration is the sheath size that will be required for each of the atherectomy devices, with the majority requiring either 6 or 7 F. Filter placement can be challenging, too, if both the SFA and DFA are patent. Filters are not required in orbital atherectomy but have been widely used in directional atherectomy. It is important to consider where the filter shall be placed in cases of multiple occlusions at the femoral bifurcation. In cases of CFA occlusion, the filter can either be placed in the SFA or the DFA. In lesions of the SFA and ostial CFA, two filters may be placed alternately, depending on the cutting direction. For CFA and SFA or CFA and DFA lesions, the filter may either be placed in the SFA or DFA, depending on the lesion. Post-dilatation is generally performed after atherectomy to obtain an optimal lumen gain and can either be performed using POBA [47]. 

##### Clinical Evidence

No trials comparing atherectomy to open surgery in CFA disease have so far been conducted. Randomised controlled trials have been limited to comparing different endovascular techniques [44,45,49,50,51]. Wardle et al. analysed data published till 2019 in a Cochrane Review and found that there is insufficient evidence demonstrating the superiority of atherectomy to POBA concerning patency, periprocedural morbidity, and mortality, regardless of stenting, in femoropopliteal disease [45]. This was recently reconfirmed in a meta-analysis where data till 2021 were scrutinised [51]. Trials focussing solely on the treatment of CFA with atherectomy are limited. Procedural complication rates comprising peripheral embolisation, vessel perforation, and access complications are reported in up to 12% of patients. Primary patency rates at one year have been described to be above 88%, with variable target revascularisation rates that go up to 14% at one year [23,44,46,50]. 

According to the subgroup analysis provided by Bonvini et al., 96% of 25 patients undergoing CFA atherectomy via SilverHawk^TM^ were successfully treated. The primary patency rate was 88.2% at one year and the target lesion revascularisation (TLR) was 4.8%. The clinical outcomes of atherectomy were superior to PTA irrespective of supplementary stenting [23,44,46]. Mehta et al. presented data from 167 patients who underwent endovascular CFA repair. Thirty-two of these patients received atherectomy after POBA via Jetstream/Pathway atherectomy (Boston Scientific, Marlborough, MA, USA). After 20 months, primary patency was 92.3% for patients treated with atherectomy compared to 72% for patients treated solely with POBA [44]. In a larger, retrospective analysis by Böhme et al., 250 patients underwent directional atherectomy of the CFA. During a median follow-up of 25 months, the rate of freedom from clinically driven TLR was 86.4%. Importantly, 10.4% of patients experienced periprocedural complications, which included access site complications, perforation of the CFA, and peripheral embolisation. All complications were treated by endovascular means, except for one case. At 30 days, 0.4% of patients experienced major adverse limb events, major adverse cardiac events, and mortality. The atherectomy catheters used, in descending order, were TurboHawk^TM^, SilverHawk^TM^, and HawkOne^TM^ [46]. A prospective, multicentre, observational study on the treatment of chronic CFA bifurcation occlusion (EAST-CFA trial, NCT05603546) has been recruiting patients.

#### 3.7.3. Intravascular Lithotripsy (IVL)

##### General Aspects

Another option for managing a high calcification burden is lithoplasty, which is based on intravascular lithotripsy. This technique uses pulsatile sonic waves, which are emitted through a traditional balloon catheter to fracture intimal and medial calcification. With this circumferential released energy, disruption of calcified atherosclerotic disease has been shown to result in lumen gain and an increase in vessel compliance by restoring vessel elasticity. This technique can only be used in calcified lesions and either as a vessel preparation tool or as a standalone procedure. Low periprocedural complication rates have been delineated. Disadvantages include challenges in reimbursement and added cost, though cost-effectiveness analyses compared to open CFA revascularisation have not been performed yet. Long-term data are lacking to date [49,52,53,54,55,56,57,58]. 

##### Technical Considerations

To perform the procedure, a 10% oversized catheter to the diameter of the reference vessel should be selected. There are two classes of balloon catheters available from the only peripheral lithoplasty system currently in use (Shockwave Medical, Fremont, CA, USA): a low-profile system S4 (2.5 to 4 mm × 40 mm) and a higher profile system M^5+^. The latter is better suited for the CFA, with balloons ranging from 3.5 to 8.0 mm × 60 mm, and will require a 6 or 7 French sheath for introduction [52,59]. Recently, the L6 has been introduced, with balloon diameters ranging between 8.0 and 12.0 mm with a length of 30 mm. These could enable adequate sizing for group I to II lesions and will soon be available on the market in Europe. Before using IVL, successful crossing after obtaining vascular access must be ensured. The balloon-based catheter is inflated with low pressures between 2 and 4 atmospheres and completed with low-pressure dilation at 6 atmospheres. No filter is required for IVL [52,59]. 

##### Clinical Evidence

To date, several trials have highlighted the safety and efficacy of IVL, which was initially investigated through the DISRUPT PAD I trial [60]. Recently, Wong et al. have analysed the existing literature in their systematic review and meta-analysis and demonstrated an almost two-thirds reduction in stenosis diameter in lower limb revascularisation, with low complication rates that were flow-limiting < 1.5% of cases [60]. Data on CFA revascularisation through IVL have been limited [49,58,61]. Brodmann et al. reported data from 21 patients from three sites who were treated with IVL for CFA stenoses with a lumen gain of an average of 3.1 mm (range from 0.7 to 5.2 mm). This resulted in a mean residual diameter stenosis of 21.3%, resembling CFA data from the DISRUPT III trial. Complications were limited to five non-flow-limiting dissections, with no perforation or embolisation experienced [54,59]. Similar data have been described in a retrospective analysis by Baig et al. including an 18-month follow-up. Clinically driven TLR reached 80.6% in the follow-up. This did not prove to be dependent on the used adjunctive therapy, though data are limited to 54 CFA lesions [55]. The DISRUPT PAD III trial has disclosed the distribution and further analysis of treated peripheral arteries, showing that the CFA was treated in 27 patients with different adjunctive therapies. Further subgroup analysis could be conducted for the DISRUPT PAD III trial and the data set from Salazar et al. [54,56]. A prospective, multicentre, single-arm trial (FESTIVAL, NCT05821829) and another single-arm trial (CRUSH PAD) have been recruiting patients for CFA endovascular repair via lithotripsy to evaluate short-term safety and efficacy.

Table 1 gives an overview of the results of trials that involved treating CFA lesions with either atherectomy or intravascular lithotripsy.

### 3.8. Antirestenotic Therapy

#### 3.8.1. Drug-Coated Balloon Angioplasty

To reduce the risk of restenosis after balloon dilation, balloons which are coated with antiproliferative medication, such as sirolimus or paclitaxel, have been developed. They aim to prolong patency by inhibiting smooth muscle hyperproliferation with neo-intimal development but will need a proper vessel preparation technique that reduces the stenotic burden below 30% and flow-limiting dissections. More evidence on the use of paclitaxel-coated devices exists, while the use of sirolimus-coated devices is currently being evaluated. Data on CFA revascularisation using paclitaxel-coated balloons have shown superior primary patency rates while having a similar postprocedural risk compared to POBA. Freedom from TLR and amputation are the same for both treatment modalities [51]. Previous studies that were indicative of higher mortality rates associated with paclitaxel-coated devices have been invalidated by studies with a higher number of enrolled patients and updated meta-analyses [65,66]. In addition, cost-effectiveness analyses have encouraged their use [67,68]. Severe calcification, as found in most CFA lesions, has been highlighted as a negative predictor of technical success within endovascular revascularisation with an elevated risk of amputation [1,52]. More importantly, calcifications may affect drug penetration into the vessel wall and decrease the efficacy of drug-coated devices [69]. Specific vessel preparation tools and atherectomy for superficial and IVL for deep calcium load treatment are of probable utility here. Both treatment options have been used together with drug-coated balloon (DCB) angioplasty.

#### 3.8.2. Stenting

##### General Aspects

Stenting the CFA has controversially been discussed, but recently gained more acceptance with technical developments in the provided stent options. Self-expanding stents are recommended to treat lesions sparing the bifurcation, while balloon-expandable stents are preferred to treat lesions involving the SFA and DFA, since they can be modified in situ to accommodate the bifurcation. If the SFA is occluded and is not aimed for revascularisation, a self-expanding stent can be placed reaching from the CFA to the DFA. A disadvantage of using balloon-expandable stents is that they cannot be punctured again and can be modified with external compressions, which is why we favour self-expanding stents. There are many different self-expandable stents to choose from [70]. As a vascular mimetic stent, the self-expanding interwoven nitinol Supera Peripheral Stent (Abbott Vascular, Santa Clara, CA, USA) offers a technically well-suited option for CFA repair. This stent is known to be flexible and to a smaller extent susceptible to stent fracture, which both were concerns in stenting of the CFA. Flexibility is relevant for long lesions. Stent modification due to external compression is limited by crush resistance ability. This is further supplemented in Supera stents by their high radial strength, which aids in treating eccentric lesions. The belief that the inguinal crease is the folding point in movement and could thus pose another difficulty has been disproven. Research has shown that the folding point in hip flexion is located more cranially and moves further up as people age [17]. An additional advantage of the Supera stent is that flow into covered arteries can be preserved as it is not covered and that future endovascular interventions are not affected by previous stent placement. This can be evaded through a short stent length, limiting the treatment to the area with the highest burden of disease. Nonetheless, vascular mimetic stents can be punctured for future interventions, which cannot be performed in balloon-expandable stents. Data on the safety of secondary puncture have recently been provided. In addition, other accesses can be used to circumvent this issue. A disadvantage is the current size limit of 7.5 mm [71,72].

##### Technical Considerations

CFA lesions that do not involve the bifurcation represent simple lesions (group I, group II within the Azéma classification) which require localised revascularisation without branched techniques. For complex revascularisation, such as CFA lesions involving the femoral bifurcation, different stenting techniques can be implemented. Most of these techniques derive from the coronary field [73]. Here, we present the main stenting techniques to preserve the bifurcation and propose a guide for their use.

##### Simple Stenting Technique

In lesions affecting solely the CFA or adjacent external iliac artery (EIA), a simple stenting technique can be used with a stent ranging from the CFA to the EIA. To avoid branch coverage in strongly developed side branches, uncovered self-expandable stents, preferably the interwoven nitinol Supera stents, are favoured over covered stents.

##### Kissing Stenting Technique

In SFA and DFA disease, the kissing stent technique can be applied. Here, two stents are implanted together by advancing one stent into the DFA and one into the SFA. At the bifurcation level, direct contact between the two stents must be achieved, enabling a slight parallel extension into the CFA. We find this technique appropriate for lesions of the bifurcation not extending too much into the CFA itself.

##### Stenting with Fenestration of the Healthy Ostium

This technique is useful when the lesion involves the CFA and extends to the SFA or the DFA but not both. A balloon-expandable stent is inflated from the CFA to the diseased branch. Its diameter is chosen according to the one of the diseased branches. The proximal stent part is overinflated to accommodate the CFA diameter. This is known as the proximal optimisation technique (POT). Then, the struts are crossed to enter the healthy artery and a balloon is inflated to open the struts and avoid stenting of this healthy branch. A final kissing balloon technique can help obtain an optimal result.

##### T and Small Protrusion (TAP) Technique

This technique directly derives from the coronary field. The TAP technique can be used when the lesion involves the CFA and extends to the SFA and the DFA. The initial steps are equivalent to the fenestration technique described above. The difference is that after the POT, the struts are crossed to enter the unstented artery and a stent is inflated to open the struts and treat the lesion. A final kissing balloon technique can help obtain an optimal result.

##### Culotte Technique

**Figure 3:**
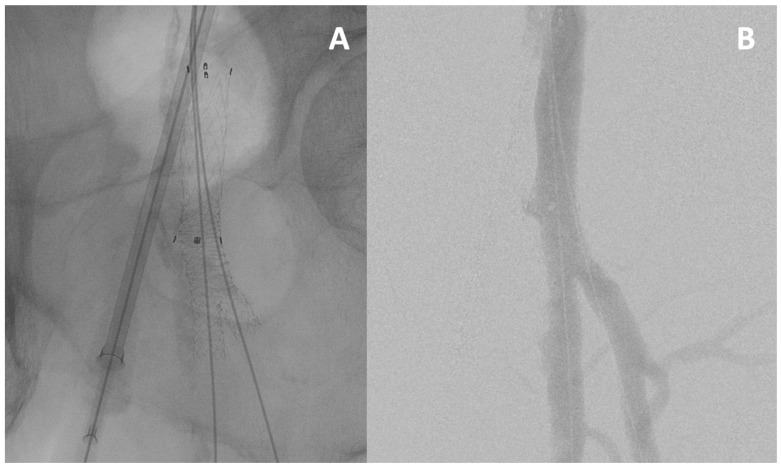
**The culotte technique**. The culotte technique is used for a complex lesion of the common femoral artery and its bifurcation, visible on fluoroscopy (**A**) and angiography (**B**).

This technique allows for the creation of a real bifurcated stent in cases of CFA, DFA, and SFA impairment (Figure 3). It starts with the TAP technique, but the protrusion of the second stent into the first is very important. A POT is performed on the second stent and its struts are crossed to end with a kissing balloon technique.

##### Clinical Evidence

In 2011, Bonvini et al. published the outcomes of 360 endovascular revascularisations of the CFA with or without adjacent arteries. The study found that the procedural-related complication rate was above 5%. Multivariate analyses of these retrospectively analysed data highlighted using stents as the only independent protective factor against procedural failure, TLR, and 1-year restenosis rate. A decline in TLR was also present when atherectomy devices were utilised.

The TECCO trial (Traitement des Lésions Athéromateuses de l’Artère Fémorale Commune par Technique Endovasculaire Versus Chirurgie Ouverte) showed no significant differences regarding one-year primary patency, clinical improvement, and target lesion revascularisation in EVT and CFA endarterectomy. Periprocedural complication rates and hospital stays were higher in OSR (26% and 6.3 days ± 3 days) versus EVT (12.5% and 3.2 ± 2.9 days) [38].

In a systematic review by Changal et al., the literature on open and endovascular CFA repair was analysed. The primary patency of routine stenting of the CFA was described at 84% (ranging between 75 and 92%) and with a median low local complication rate of above 5%. The most common complication in the routine stenting group was in-stent restenosis, which was observed in 10.4% of cases. This was followed by stent fracture (1.9%), haematoma (0.65%), stent occlusion (0.65%), and inflow or outflow vessel stenosis (0.65%), in descending order. Haematoma was observed almost five times more often in selective stenting (3.2%). Systemic complications were more often observed after open surgical repair [74]. Two prospective randomised controlled trials are investigating the safety and efficacy of endovascular CFA repair with Supera Peripheral Stents: these are the VMI-CFA (NCT-02804113) and SUPERSURG (NCT-04349657) trials. Interim analyses that have been presented at conferences have shown a primary patency of over 90% at two years.

### 3.9. Treatment Algorithm

**Figure 4:**
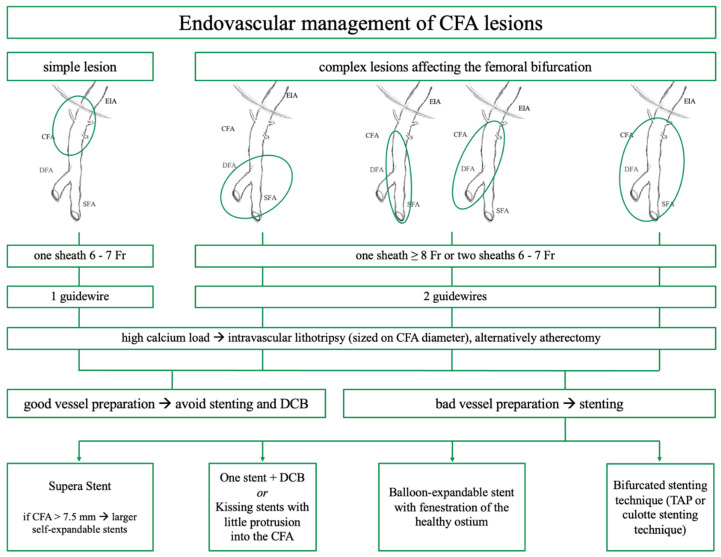
Treatment algorithm of endovascular treatment of common femoral artery (CFA) lesions. The presented treatment algorithm for the management of CFA lesions is based on disease extent and lesion characteristics. In cases of stenting, the therapy is delineated according to the lesion found in the same column. It ranges from simple stenting via the Supera stent, stenting with drug-coated balloon angioplasty (DCB) or kissing stents, and balloon-expandable stenting with fenestration to bifurcated stenting, where T and small protrusion (TAP) or culotte stenting can be applied.

Based on the current knowledge and experience, we hereby present a treatment algorithm for EVT of CFA atherosclerotic disease (Figure 4).

## 4. Conclusions and Perspectives

There is a lack of large-scale high-quality data comparing the clinical course and cost-effectiveness of open and endovascular surgical repair of the CFA. As a result, the treatment choice is dependent on subjective factors, such as the department, physician preferences, and reimbursement systems, in addition to patient characteristics. To surpass the barriers to the design and delivery of such trials, potential obstacles have been delineated through a mixed-methods qualitative study based on physicians’ experience and opinions [75]. We encourage performing randomised controlled trials based on the recommended aspects complemented by reporting standards for an improved comparison between the delineated treatment options and to supplement them with patient-reported outcomes.

## Figures and Tables

**Table 1:** Summary of studies that present atherectomy and intravascular lithotripsy as vessel preparation tools in the common femoral artery (CFA).

Authors	Publication Year	Trial	CFA Lesions (n)	Preparation Tool	Devices Used	DCB	Filter	Technical Success	Complications n (%)	Bailout Stenting n (%)	Freedom from TLR
Bonvini [23]	2011	retrospective, registry, subgroup	25	atherectomy	SH	NR	NR	96%	0	NR	95.2%
Lee [48]	2017	retrospective, registry, subgroup	200	atherectomy	NR	NR	NR	NR	34 (17)	12 (6)	NR
Stavroulakis [50]	2018	retrospective, subgroup	21	atherectomy	TH, HO, P	100%	100%	95%	6 (28.6)	1 (4.8)	89%
Picazo [62]	2020	retrospective	25	atherectomy	HO	92%	100%	92%	3 (12)	1 (4)	93.4%
Böhme [46]	2020	retrospective	250	atherectomy	TH, HO, SH	60.4%	75.6%	92.4%	26 (10.4)	20 (8)	86.4% *
Cioppa [47]	2021	retrospective, registry	80	atherectomy	TH, SH	100%	100%	100%	0	6 (7.5)	86.7%
Baig [49]	2022	retrospective, subgroup	35	atherectomy	SH, HO, DB	100%	83%	100%	2 (5.7)	2 (5.7)	91.2%
Baig [49]	2022	retrospective, subgroup	33	intravascular lithotripsy	Shockwave Medical	100%	0	100%	1 (3)	0	79.4%
Stavroulakis [61]	2023	retrospective	33	intravascular lithotripsy	Shockwave Medical	90%	NR	97%	4 (12)	4 (12)	94%

This is a list of studies with at least 20 lesions treated with either atherectomy or intravascular lithotripsy, supplemented by drug-coated balloon angioplasty (DCB). SilverHawk (SH), TurboHawk (TH), HawkOne (HO), Pantheris (P), and Diamondback 360 (DB) are the atherectomy devices named within these studies. For intravascular lithotripsy, the only device on the market is represented by Shockwave Medical. Periprocedural information is provided and includes freedom from target lesion revascularisation (TLR) of patients reaching follow-up at one year (* for a prolonged follow-up period). In cases of missing information, this is indicated by NR, standing for not reported. Trials with low patient recruitments, different combinations of treatment modalities, and/or those lacking a subgroup analysis were excluded [54,56,59,63,64].

## References

[1] Conte M.S., Bradbury A.W., Kolh P., White J.V., Dick F., Fitridge R., Mills J.L., Ricco J.-B., Suresh K.R., Murad M.H. (2019). Global vascular guidelines on the management of chronic limb-threatening ischemia. J. Vasc. Surg..

[2] Nordanstig J., Behrendt C.-A., Baumgartner I., Belch J., Bäck M., Fitridge R., Hinchliffe R., Lejay A., Mills J.L., Rother U. (2024). Editor’s Choice- European Society for Vascular Surgery (ESVS) 2024 Clinical Practice Guidelines on the Management of Asymptomatic Lower Limb Peripheral Arterial Disease and Intermittent Claudication. Eur. J. Vasc. Endovasc. Surg..

[3] Halpin D., Erben Y., Jayasuriya S., Cua B., Jhamnani S., Mena-Hurtado C. (2017). Management of Isolated Atherosclerotic Stenosis of the Common Femoral Artery: A Review of the Literature. Vasc. Endovasc. Surg..

[4] Quax M.L.J., Eefting D., Smeets H.J. (2021). Determinants of Success and Early Complications in Common Femoral Artery Endarterectomy: A Retrospective Study. Surgeries.

[5] Derksen W.J.M., Verhoeven B.A.N., van de Mortel R.H.W., Moll F.L., de Vries J.-P.P.M. (2009). Risk Factors for Surgical-Site Infection Following Common Femoral Artery Endarterectomy. Vasc. Endovasc. Surg..

[6] Kuma S., Tanaka K., Ohmine T., Morisaki K., Kodama A., Guntani A., Ishida M., Okazaki J., Mii S. (2016). Clinical Outcome of Surgical Endarterectomy for Common Femoral Artery Occlusive Disease. Circ. J..

[7] Hashimoto T., Yamamoto S., Kimura M., Sano M., Sato O., Deguchi J. (2022). Long-Term Outcomes following Common Femoral Endarterectomy. J. Clin. Med..

[8] Siracuse J.J., Gill H.L., Schneider D.B., Graham A.R., Connolly P.H., Jones D.W., Meltzer A.J. (2014). Assessing the Perioperative Safety of Common Femoral Endarterectomy in the Endovascular Era. Vasc. Endovasc. Surg..

[9] Uhl C., Goetzke H., Woronowicz S., Betz T., Toepel I., Steinbauer M. (2020). Treatment of Lymphatic Complications after Common Femoral Artery Endarterectomy. Ann. Vasc. Surg..

[10] Springhorn M.E., Kinney M., Littooy F.N., Saletta C., Greisler H.P. (1991). Inflow Atherosclerotic Disease Localized to the Common Femoral Artery: Treatment and Outcome. Ann. Vasc. Surg..

[11] Linni K., Ugurluoglu A., Hitzl W., Aspalter M., Hölzenbein T. (2014). Bioabsorbable Stent Implantation vs. Common Femoral Artery Endarterectomy: Early Results of a Randomized Trial. J. Endovasc. Ther..

[12] Nguyen B.-N., Amdur R.L., Abugideiri M., Rahbar R., Neville R.F., Sidawy A.N. (2015). Postoperative complications after common femoral endarterectomy. J. Vasc. Surg..

[13] Chaney M., Joshi G., Serrato J.C., Rashid M., Jacobs A., Jacobs C.E., White J.V., Schwartz L.B., El Khoury R. (2024). Morbidity and mortality of common femoral endarterectomy. J. Vasc. Surg..

[14] Herisson F., Heymann M.-F., Chétiveaux M., Charrier C., Battaglia S., Pilet P., Rouillon T., Krempf M., Lemarchand P., Heymann D. (2011). Carotid and femoral atherosclerotic plaques show different morphology. Atherosclerosis.

[15] Rabellino M., Di Caro V., Raleigh J.V., Jose C., Vadim K., Maynar M., Zander T. (2021). Common Femoral Artery Stenting: Computed Tomography Angiography Based Long-Term Patency. Vasc. Endovasc. Surg..

[16] Lechner G., Jantsch H., Waneck R., Kretschmer G. (1988). The relationship between the common femoral artery, the inguinal crease, and the inguinal ligament: A guide to accurate angiographic puncture. Cardiovasc. Interv. Radiol..

[17] Park S.I., Won J.H., Kim B.M., Kim J.K., Lee D.Y. (2005). The Arterial Folding Point During Flexion of the Hip Joint. Cardiovasc. Interv. Radiol..

[18] Aboyans V., Ricco J.-B., Bartelink M.-L.E.L., Björck M., Brodmann M., Cohnert T., Naylor A.R., Roffi M., Tendera M., Vlachopoulos C. (2018). Editor’s Choice—2017 ESC Guidelines on the Diagnosis and Treatment of Peripheral Arterial Diseases, in collaboration with the European Society for Vascular Surgery (ESVS). Eur. J. Vasc. Endovasc. Surg..

[19] Swanberg J., Nyman R., Magnusson A., Wanhainen A. (2014). Selective Intra-arterial Dual-energy CT Angiography (s-CTA) in Lower Extremity Arterial Occlusive Disease. Eur. J. Vasc. Endovasc. Surg..

[20] Rofsky N.M., Adelman M.A. (2000). MR Angiography in the Evaluation of Atherosclerotic Peripheral Vascular Disease. Radiology.

[21] Makris G.C., Chrysafi P., Little M., Patel R., Bratby M., Wigham A., Anthony S., Uberoi R. (2017). The role of intravascular ultrasound in lower limb revascularization in patients with peripheral arterial disease. Int. Angiol..

[22] Smith J.A., Yang L., Chen L., Kumins N., Cho J.S., Harth K., Wong V., Kashyap V., Colvard B. (2023). Trends and outcomes associated with intravascular ultrasound use during femoropopliteal revascularization in the Vascular Quality Initiative. J. Vasc. Surg..

[23] Bonvini R.F., Rastan A., Sixt S., Noory E., Schwarz T., Frank U., Roffi M., Dorsaz P.A., Schwarzwälder U., Bürgelin K. (2011). Endovascular Treatment of Common Femoral Artery Disease: Medium-Term Outcomes of 360 Consecutive Procedures. J. Am. Coll. Cardiol..

[24] Azéma L., Davaine J., Guyomarch B., Chaillou P., Costargent A., Patra P., Gouëffic Y. (2011). Endovascular Repair of Common Femoral Artery and Concomitant Arterial Lesions. Eur. J. Vasc. Endovasc. Surg..

[25] Rabellino M., Raleigh J.V., Chiabrando J.G., Di Caro V., Chas J., Garagoli F., Bluro I. (2022). Novel Common Femoral Artery Lesion Classification in Patients Undergoing Endovascular Revascularization. Cardiovasc. Interv. Radiol..

[26] Stoner M.C., Calligaro K.D., Chaer R.A., Dietzek A.M., Farber A., Guzman R.J., Hamdan A.D., Landry G.J., Yamaguchi D.J. (2016). Reporting standards of the Society for Vascular Surgery for endovascular treatment of chronic lower extremity peripheral artery disease. J. Vasc. Surg..

[27] Noori V.J., Eldrup-Jorgensen J. (2018). A systematic review of vascular closure devices for femoral artery puncture sites. J. Vasc. Surg..

[28] Dudeck O., Teichgraeber U., Podrabsky P., Haenninen E.L., Soerensen R., Ricke J. (2004). A Randomized Trial Assessing the Value of Ultrasound-Guided Puncture of the Femoral Artery for Interventional Investigations. Int. J. Cardiovasc. Imaging.

[29] Seto A.H., Abu-Fadel M.S., Sparling J.M., Zacharias S.J., Daly T.S., Harrison A.T., Suh W.M., Vera J.A., Aston C.E., Winters R.J. (2010). Real-Time Ultrasound Guidance Facilitates Femoral Arterial Access and Reduces Vascular Complications: FAUST (Femoral Arterial Access with Ultrasound Trial). JACC Cardiovasc. Interv..

[30] Irani F., Kumar S., Colyer W.R. (2009). Common femoral artery access techniques: A review. J. Cardiovasc. Med..

[31] Avraham E., Natour M., Obaid W., Karmeli R. (2020). Superficial femoral artery access for endovascular aortic repair. J. Vasc. Surg..

[32] Dolan J. (2020). The superficial femoral artery: A novel site for arterial access. Br. J. Anaesth..

[33] Gutzeit A., van Schie B., Schoch E., Hergan K., Graf N., Binkert C.A. (2012). Feasibility and Safety of Vascular Closure Devices in an Antegrade Approach to Either the Common Femoral Artery or the Superficial Femoral Artery. Cardiovasc. Interv. Radiol..

[34] Gutzeit A., Schoch E., Sautter T., Jenelten R., Graf N., Binkert C.A. (2010). Antegrade Access to the Superficial Femoral Artery with Ultrasound Guidance: Feasibility and Safety. J. Vasc. Interv. Radiol..

[35] Gutzeit A., Graf N., Schoch E., Sautter T., Jenelten R., Binkert C.A. (2011). Ultrasound-guided antegrade femoral access: Comparison between the common femoral artery and the superficial femoral artery. Eur. Radiol..

[36] Kennedy S.A., Rajan D.K., Bassett P., Tan K.T., Jaberi A., Mafeld S. (2021). Complication rates associated with antegrade use of vascular closure devices: A systematic review and pooled analysis. J. Vasc. Surg..

[37] Franz R.W., Tanga C.F., Herrmann J.W. (2017). Treatment of peripheral arterial disease via percutaneous brachial artery access. J. Vasc. Surg..

[38] Gouëffic Y., Della Schiava N., Thaveau F., Rosset E., Favre J.-P., du Mont L.S., Alsac J.-M., Hassen-Khodja R., Reix T., Allaire E. (2017). Stenting or Surgery for De Novo Common Femoral Artery Stenosis. JACC Cardiovasc. Interv..

[39] Baumann F., Ruch M., Willenberg T., Dick F., Do D.-D., Keo H.-H., Baumgartner I., Diehm N. (2011). Endovascular treatment of common femoral artery obstructions. J. Vasc. Surg..

[40] Patrone L., Ysa A., Covani M., Lichaa H. (2023). Antegrade Crossing Techniques for Hard Proximal Occlusion Caps without the Use of Dedicated Chronic Total Occlusion Devices: A Pictorial Review. J. Endovasc. Ther..

[41] Lorenzoni R., Ferraresi R., Manzi M., Roffi M. (2015). Guidewires for lower extremity artery angioplasty: A review. EuroIntervention.

[42] Chen W., Labropoulos N., Pacanowski J., Leon L.R. (2020). Retrograde deep femoral artery puncture for the treatment of an iatrogenic dissection flap of the common femoral artery bifurcation. J. Vasc. Surg. Cases Innov. Tech..

[43] Dacie J.E., Tennant D. (1990). A new approach to percutaneous transluminal angioplasty of profunda femoris origin stenosis. Cardiovasc. Interv. Radiol..

[44] Mehta M., Zhou Y., Paty P.S., Teymouri M., Jafree K., Bakhtawar H., Hnath J., Feustel P. (2016). Percutaneous common femoral artery interventions using angioplasty, atherectomy, and stenting. J. Vasc. Surg..

[45] Wardle B.G., Ambler G.K., Radwan R.W., Hinchliffe R.J., Twine C.P. (2020). Atherectomy for peripheral arterial disease. Cochrane Database Syst. Rev..

[46] Böhme T., Romano L., Macharzina R.-R., Noory E., Beschorner U., Jacques B., Bürgelin K., Flügel P.-C., Zeller T., Rastan A. (2021). Outcomes of directional atherectomy for common femoral artery disease. EuroIntervention.

[47] Cioppa A., Franzese M., Gerardi D., Pucciarelli A., Popusoi G., Stabile E., Salemme L., Sada L., Verdoliva S., Burattini O. (2022). Three-year outcome of directional atherectomy and drug coated balloon for the treatment of common femoral artery steno-occlusive lesions. Catheter. Cardiovasc. Interv..

[48] Lee M.S., Heikali D., Mustapha J., Adams G., Mahmud E. (2017). Acute procedural outcomes of orbital atherectomy for the treatment of common femoral artery disease: Sub-analysis of the CONFIRM Registries. Vasc. Med..

[49] Baig M., Kwok M., Aldairi A., Imran H.M., Khan M.S., Ngmadu K.S., Hyder O.N., Aronow H.D., Soukas P.A. (2022). Intravascular Lithotripsy vs Atherectomy in the Treatment of Calcified Common Femoral Artery Disease. J. Soc. Cardiovasc. Angiogr. Interv..

[50] Stavroulakis K., Schwindt A., Torsello G., Beropoulis E., Stachmann A., Hericks C., Bollenberg L., Bisdas T. (2018). Directional Atherectomy with Antirestenotic Therapy vs Drug-Coated Balloon Angioplasty Alone for Common Femoral Artery Atherosclerotic Disease. J. Endovasc. Ther..

[51] Koeckerling D., Raguindin P.F., Kastrati L., Bernhard S., Barker J., Centeno A.C.Q., Raeisi-Dehkordi H., Khatami F., Niehot C., Lejay A. (2023). Endovascular revascularization strategies for aortoiliac and femoropopliteal artery disease: A meta-analysis. Eur. Hear. J..

[52] Nasiri A., Kim H., Gurusamy V., Benenati J.F. (2022). Management of Calcification: Rational and Technical Considerations for Intravascular Lithotripsy. Tech. Vasc. Interv. Radiol..

[53] Brodmann M., Werner M., Brinton T.J., Illindala U., Lansky A., Jaff M.R., Holden A. (2017). Safety and Performance of Lithoplasty for Treatment of Calcified Peripheral Artery Lesions. J. Am. Coll. Cardiol..

[54] Adams G., Shammas N., Mangalmurti S., Bernardo N.L., Miller W.E., Soukas P.A., Parikh S.A., Armstrong E.J., Tepe G., Lansky A. (2020). Intravascular Lithotripsy for Treatment of Calcified Lower Extremity Arterial Stenosis: Initial Analysis of the Disrupt PAD III Study. J. Endovasc. Ther..

[55] Baig M., Kwok M., Aldairi A., Imran H.M., Khan M.S., Moustafa A., Hyder O.N., Saad M., Aronow H.D., Soukas P.A. (2022). Endovascular Intravascular Lithotripsy in the Treatment of Calcific Common Femoral Artery Disease: A Case Series with an 18-Month Follow-Up. Cardiovasc. Revascularization Med..

[56] Salazar S.A., Vengalasetti Y., Kilbridge M., Gurusamy V., Powell A., Schiro B.J., Peña C.S., Gandhi R.T., Niekamp A.S. (2023). Outcomes of Intravascular Lithotripsy in the Treatment of Chronic Limb-Threatening Ischemia: A Single-Center Retrospective Study. Cardiovasc. Interv. Radiol..

[57] Wong C.P., Chan L.P., Au D.M., Chan H.W.C., Chan Y.C. (2022). Efficacy and Safety of Intravascular Lithotripsy in Lower Extremity Peripheral Artery Disease: A Systematic Review and Meta-Analysis. Eur. J. Vasc. Endovasc. Surg..

[58] Trani C., Russo G., Aurigemma C., Burzotta F. (2019). The conundrum of endovascular common femoral artery treatment: A case report of lithoplasty as a viable solution. Eur. Hear. J. Case Rep..

[59] Brodmann M., Schwindt A., Argyriou A., Gammon R. (2019). Safety and Feasibility of Intravascular Lithotripsy for Treatment of Common Femoral Artery Stenoses. J. Endovasc. Ther..

[60] Wong G., Lahsaei S., Aoun J., Garcia L.A. (2019). Management of common femoral artery occlusive disease: A review of endovascular treatment strategies and outcomes. Catheter. Cardiovasc. Interv..

[61] Stavroulakis K., Torsello G., Chlouverakis G., Bisdas T., Damerau S., Tsilimparis N., Argyriou A. (2023). Intravascular Lithotripsy and Drug-Coated Balloon Angioplasty for Severely Calcified Common Femoral Artery Atherosclerotic Disease. J. Endovasc. Ther..

[62] Picazo F., Kwok R.C., Hockley J.A., Garbowski M.W., Samuelson S.D., Jansen S.J. (2020). Directional Atherectomy of the Common Femoral Artery: Complications and Outcomes. Ann. Vasc. Surg..

[63] Shammas N.W., Shammas G.A., Karia R., Khalafallah R., Jones-Miller S., Shammas A.N. (2021). Two-Year Outcomes of Endovascular Interventions of the Common Femoral Artery: A Retrospective Analysis from Two Medical Centers. Cardiovasc. Revascularization Med..

[64] Dattilo P.B., Tsai T.T., Rogers R.K., Casserly I.P. (2013). Acute and medium-term outcomes of endovascular therapy of obstructive disease of diverse etiology of the common femoral artery. Catheter. Cardiovasc. Interv..

[65] Nordanstig J., James S., Andersson M., Andersson M., Danielsson P., Gillgren P., Delle M., Engström J., Fransson T., Hamoud M. (2020). Mortality with Paclitaxel-Coated Devices in Peripheral Artery Disease. N. Engl. J. Med..

[66] Dinh K., Limmer A.M., Chen A.Z.L., Thomas S.D., Holden A., Schneider P.A., Varcoe R.L. (2021). Mortality Rates after Paclitaxel-Coated Device Use in Patients with Occlusive Femoropopliteal Disease: An Updated Systematic Review and Meta-Analysis of Randomized Controlled Trials. J. Endovasc. Ther..

[67] Katsanos K., Geisler B.P., Garner A.M., Zayed H., Cleveland T., Pietzsch J.B. (2016). Economic analysis of endovascular drug-eluting treatments for femoropopliteal artery disease in the UK. BMJ Open.

[68] Sridharan N.D., Boitet A., Smith K., Noorbakhsh K., Avgerinos E., Eslami M.H., Makaroun M., Chaer R. (2019). Cost-effectiveness analysis of drug-coated therapies in the superficial femoral artery. J. Vasc. Surg..

[69] Fanelli F., Cannavale A., Gazzetti M., Lucatelli P., Wlderk A., Cirelli C., D’adamo A., Salvatori F.M. (2014). Calcium Burden Assessment and Impact on Drug-Eluting Balloons in Peripheral Arterial Disease. Cardiovasc. Interv. Radiol..

[70] Stricker H., Spinedi L., Limoni C., Giovannacci L. (2020). Stent-Assisted Angioplasty (SAA) at the Level of the Common Femoral Artery Bifurcation: Long-Term Outcomes. Cardiovasc. Interv. Radiol..

[71] Horie K., Takahara M., Nakama T., Tobita K., Tanaka A., Shintani Y., Tsubakimoto Y., Yoshioka N., Hayakawa N., Sasaki S. (2024). Multicenter Registry of Common Femoral Artery Disease Treated with Endovascular Revascularization Using Interwoven Nitinol Stents: An Observational Retrospective Study. J. Endovasc. Ther..

[72] Tao M.J., Gotra A., Tan K.T., Eisenberg N., Roche-Nagle G., Mafeld S. (2022). SUPERA Stenting in the Common Femoral Artery: Early Experience and Practical Considerations. Vasc. Endovasc. Surg..

[73] Raphael C.E., O’kane P.D. (2021). Contemporary approaches to bifurcation stenting. JRSM Cardiovasc. Dis..

[74] Changal K.H., Syed M.A., Dar T., Mangi M.A., Sheikh M.A. (2019). Systematic Review and Proportional Meta-Analysis of Endarterectomy and Endovascular Therapy with Routine or Selective Stenting for Common Femoral Artery Atherosclerotic Disease. J. Interv. Cardiol..

[75] Kaneta G., Saratzis A., Zayed H. (2023). Perception and acceptability of open versus endovascular treatment of common femoral artery disease: Barriers and facilitators for randomised controlled trials. J. Vasc. Soc. G. B. Irel..

